# The Problem of the Poor

**Published:** 1895-01-19

**Authors:** 


### The Hospital

An Institutional Journal of

The Medical Sciences and Hospital Administration, with

AN EXTRA NURSING SUPPLEMENT.

Vol. XVII.—No. 434.] [Jan. 19, 1895.

### THE PROBLEM OF THE POOR.

The larger medicine is compelled to concern itself with the poor and their poverty, inasmuch as poverty by itself is one of the most wide-extending of all the causes of disease. Low nutrition, life spent in vitiated atmospheres, worry and care, fill, not poor-houses only nor even lunatic asylums, but hospitals and the parish doctor's visiting list. If the poor, by one great effort or by many persistent smaller efforts of the better classes, could be raised above their poverty, a service of the first order would be rendered to the State and to humanity.

We are not among those who regard the problem of the poor as being practically insoluble, or even very difficult. Those who have made "a business" and "a conscience" of dealing with the poor on a small scale have very generally had reason to be well satisfied with their results. Undoubtedly those principles which succeed on a small scale will succeed on a larger scale, on the largest, indeed, provided always that they are applied with the sympathy, conscience, and intelligence which assured their success on the smaller. In a word, we are persuaded that there are very few problems of a practical kind which a body of Englishmen cannot solve, if they will but give themselves the necessary trouble, and turn an absolutely deaf ear to those irresponsible outside criticisms which in most instances are of no more significance than a puff of vapour.

A meeting in connection with the Problem of the Poor movement was held under the auspices of the Lord Mayor in the Parlour of the Mansion House last week. Mr. Burdett, in the absence of the Lord Mayor, presided, and gave a brief outline of the actual work done by the association up to the present time. It will be remembered that the association consists entirely of "Friendly Workers," drawn from and representing all the principal religious organisations of the United Kingdom. Its general committee is composed of titular heads of those organisations. Among the representatives of churches who have taken an active part in its work are the Bishop of London, Cardinal Vaughan, the Rev. Dr. Horton, the Rev. Dr. Rigg, the Rev. Dr. Adler, and many others. The late Sir Andrew Clark, President of the Royal College of Physicians, was deeply interested in the movement, and gave freely both money and personal services, to its objects.

The meeting of Wednesday was called for the twofold object of strengthening organisation by the appointment of additional secretaries and other officials to certain departments of the work, and bringing the officers of outlying districts together so as to keep them well informed as to the present whereabouts and general operations of the association as a whole. It was in every way a successful meeting. The Chairman gave a vast mass of condensed information, and produced a scheme of extension and administration which both surprised and pleased the associates present, alike by the fullness of its knowledge, the soundness of its principles, and the practicality of its details. The scheme was accepted not only unanimously, but with enthusiasm; and a general feeling pervaded the meeting that if its associates would but have the persistency of purpose to hold firmly together, a movement had been well initiated which would grow and extend until all Great Britain had been brought within the range of its benevolent and useful activities.

For the Friendly Workers' Association for the Solution of the Problem of the Poor is above all things practical. What, in a word, has it done, and what is it now doing? The answers to these questions must be summarised in half-a-dozen sentences. It has been in full operation for more than four years in North Kensington. There it has set itself to become acquainted with every family and every member of every family among the poorer part of the population. The really needy and helpless it has fed and clothed; the workless it has provided with work; the idlers it has exposed, and to a large extent banished; and it has, at the same time, secured the grateful goodwill of the poor and the earnest co-operation of the well-to-do. This is the object lesson which the Association has provided for all who are minded to deal in a practical way with social problems.

Will a scheme which has been successful in North Kensington solve the problem of the poor in other and very different districts of London and the country? That is the question which the Bishop of London has asked, and which other practical persons every where will also ask. The question can be answered in no other way but by actual experiment. *Fiat experimentum* is as applicable in the social as in the scientific laboratory. Four experiments are now being simultaneously made in four widely different parts of London. These experiments will be watched with deep interest as the result may give a solution of the Problem of the Poor.

Can the common religion, when stripped of its accidents, apply a solution to this problem ; one which is not co-extensive with civilisation only, but is as wide as humanity ? We think it can. In any case the experiment is profoundly interesting, not from the point of view of the problem of the poor only, but from the standpoint of the survival or non-survival of religion itself.

